# Validation of the EQ-5D in Taiwan using item response theory

**DOI:** 10.1186/s12889-021-12334-y

**Published:** 2021-12-19

**Authors:** Tzu-Hung Liu, Andrew D. Ho, Yu-Tien Hsu, Chih-Cheng Hsu

**Affiliations:** 1 Department of Family Medicine, Taipei Tzu Chi Hospital, Buddhist Tzu Chi Medical Foundation, No. 289, Jianguo Road, Xindian District, New Taipei City, 231 Taiwan; 2 Department of Medical Humanities, School of Medicine, Tzu Chi University, No. 701, Section 3, Zhongyang Road, Hualien City, Hualien County 970 Taiwan; 3 Harvard Graduate School of Education, Cambridge, USA; 4 Department of Social & Behavioral Sciences, Harvard T. H. Chan School of Public Health, Boston, USA; 5 Institute of Population Health Sciences, National Health Research Institutes, Miaoli County, Taiwan

**Keywords:** Validity, EQ-5D (EuroQol-5 dimensions), NHIS (National Health Interview Survey), IRT (Item response theory), Taiwan

## Abstract

**Background:**

Our study aims to provide validity evidence for the EuroQol five dimensions questionnaire (EQ-5D) in the National Health Interview Survey of Taiwan in the 2013 wave and further interpret the EQ-5D scores for patients with chronic diseases. Another goal of the study was to use item response theory (IRT) to identify items that are informative for assessing quality of life using EQ-5D.

**Methods:**

There were 17,260 participants, aged 12-64, who completed the interviews in our study. Psychometric methods, including factor analysis and the IRT model known as the Graded Response Model (GRM), were used to assess the unidimensionality of EQ-5D and its item properties. Correlation analysis was used to assess whether EQ-5D scores are associated with scores from the 36-Item Short Form Survey (SF-36).

**Results:**

The EQ-5D scores have moderate internal consistency (Cronbach’s alpha: 0.60) and a scree plot suggests that the EQ-5D measure is unidimensional. The item information function analysis from the IRT model demonstrates that the first 3 items, “mobility,” “self-care,” and “usual activities” are the most informative items for patients who have chronic diseases and health-related quality of life below the 10th percentile. The EQ-5D scores have a moderate correlation (r: 0.61) with SF-36 scores.

**Conclusions:**

The EQ-5D scale shows promise for use in the general population. The IRT model informs our interpretation of the EQ-5D scores. Given the time constraints in clinical settings, we suggest using the first three items in EQ-5D to measure the health-related quality of life for patients with chronic diseases.

**Supplementary Information:**

The online version contains supplementary material available at 10.1186/s12889-021-12334-y.

## Background

As the health-related quality of life (HRQoL) can supplement the disease diagnosis and provide more information on disease burden, many instruments have been developed to assess the HRQoL. The EuroQol 5-dimension (EQ-5D) questionnaire is one of the highly-utilized instruments consisting of five domains: mobility, self-care, usual activities, pain/discomfort, and anxiety/depression. The questionnaire was first introduced by EuroQol Group in 1990 [1] and has been proven to be a valid instrument for the general population and patients with chronic conditions [2–6]. The 36-Item Short Form Survey (SF-36) is another widely-used HRQoL measurement in clinical research and population studies [7–9]. Many researchers used both EQ-5D and SF-36 in their studies and found the two instruments showed similar results [10–13]. Previous studies also suggested that approaches mapping the SF-36 scale onto the EQ-5D scale are robust across settings and medical conditions [14, 15]. When compared with the SF-36, the EQ-5D is advantageous to HRQoL studies because of its low cognitive demand and high efficiency. Use of the EQ-5D in large-scale interview surveys in Taiwan had been shown to be an effective and simple approach [16–18].

Item response theory (IRT) has been recognized as a useful and powerful tool for evaluation, especially for the educational test development [19]. IRT has also been increasingly applied for quality-of-life research in recent years [20–27]. In contrast to classic test theory that focuses on average test scores, IRT focuses on a single dimension or latent construct. IRT analysis estimates different item features, and item characteristics are expected to remain the same and will not change due to the sampled population. The item parameters include location and information parameters. The information parameter indicates how an item can distinguish people with different levels of ability. The location parameter shows where on the scale of the ability an item has its most discriminative information. Based on the item responses in EQ-5D, the IRT model could potentially inform us which items better distinguish between levels of quality of life. Therefore, IRT can improve the measurement accuracy by evaluating the items to a finer degree and also improve the efficiency of work by identifying the most useful items for a shortened measure.

To the best of our knowledge, there is no literature that evaluates the EQ-5D using the IRT model among the Taiwanese population. Therefore, in this study, we aim to use IRT to assess whether both relatively healthy people and patients with chronic diseases can be measured on this common HRQoL scale. Furthermore, we hope to provide validity evidence that the EQ-5D measures quality of health based on its content, shows coherence in its scores, and correlates to other HRQoL measurements such as the SF-36. Additionally, this study demonstrates how the IRT model can provide useful insights about the design of scales for quality of life.

## Methods

### Data and sample

The National Health Interview Survey (NHIS) – Taiwan is a national survey conducted jointly by Health Promotion Administration, Ministry of Health and Welfare and National Health Research Institutes, Taiwan [17]. The survey is administered every 4 years to assist Taiwan’s public health sector to monitor the health status of the population. Our data came from the NHIS conducted in 2013, which included both EQ-5D and SF-36 in its design and was the most recent data available for the purpose of our study. NHIS was approved by the ethics committee of the National Health Research Institute in Taiwan on July 24, 2013 (Code: EC1020502). The interviewees in this survey comprised a representative sample of national and city/county populations in Taiwan. A multistage stratified systematic sampling design was applied. The townships were stratified by the urbanization and location before sampling. Village/Lin in sampled townships and then the individual in sampled village/lin were selected step by step following the principle of probability proportional to size (PPS). A total of 159 interviewers were recruited and trained for this research. All interviewees signed consent forms. Data was collected using face to face interviews from July to December in 2013. There were three sets of questions designated for three age groups, including age under 12, 12 to 64, and 65 or above. Our study only selected the interviewees aged 12-64 because the questionnaires for the other two age groups did not include SF-36. The response rate was 72.2% for this age group, and 17,260 participants completed the interviews.

The contents of the questionnaire include personal characteristics, health status, chronic diseases, EQ-5D, and SF-36 (optional) for measuring the quality of life in the general population (see Supplementary material A and B for further details). The NHIS required the participants to fill EQ-5D and SF-36 themselves, including the participants aged below 18. There were no proxy respondents used in the data collection. For those who completed both EQ-5D and SF-36, they all filled SF-36 first and then EQ-5D. A total of 8,272 participants (47.9%) filled out the SF-36 forms. Since only about half of the participants finished SF-36, we conducted a sensitivity analysis to compare the sociodemographic characteristics between those filled SF-36 and those who did not. The chronic diseases registered in the NHIS catalog were hypertension, diabetes, dyslipidemia, stroke, asthma, chronic kidney disease, heart disease, gout, peptic ulcer, chronic obstructive pulmonary disease (COPD), liver/gallbladder disease, osteoporosis, cancer, osteoarthritis, psychiatric disorder, benign prostatic hyperplasia (BPH, for male only), and uterine/ovarian disease (for female only).

There are only 6 items in the EQ-5D scale, including five items in the descriptive system (each item assigned to one specific domain) and one item on a visual analogue scale (VAS). The domains include mobility (D1), self-care (D2), usual activities (D3), pain/discomfort (D4), and anxiety/depression (D5). Each domain was rated by 3 levels (1: no problem; 2: some/moderate problem; 3: unable to do/extreme problem). The average score of the first five items is the EQ-5D index. In the sixth item, the participants can respond with a score between 0 (representing the poorest health status) and 100 (representing the best health status). The SF-36 includes 36 items. These items can be summarized into the physical component score (PCS), the mental component score (MCS), and the total score [7].

### Statistical Methods

Descriptive summaries of demographic results are shown. Using classical test theory, we estimated the inter-item correlation coefficients and the Cronbach’s alpha that described internal consistency. We examined the dimensionality using factor analysis and principal component analysis. The IRT graded response model was used for estimating location and information parameters. Based on the item responses in EQ-5D, the HRQoL can also be estimated from the graded response model, and we name this the EQ-5D scale score throughout our analysis.

We calculated the EQ-5D scale score for each chronic disease. The item information functions would be presented and show how much information the items can provide and in what range of EQ-5D scale score the items are most informative. We then calculated the conditional standard error of measurement (CSEM) for EQ-5D scale score using the graded response model. CSEM measures the standard deviation of the observed scores of a survey taker with a fixed and unchanging true score over repeated measurements using these items. CSEM indicates the precision of EQ-5D scale scores at different levels, and a smaller CSEM indicates the measurement is more precise for examinees.

To gather predictive evidence, we also correlated the EQ-5D scores (EQ-5D index, EQ-5D VAS, and EQ-5D scale score) with the SF-36 scores, including the SF-36 physical component score (PCS), the SF-36 mental component score (MCS), and the SF-36 total scores. An alpha level of 0.05 was used as the cutoff for statistical significance. Stata/IC version 15.1 (StataCorp, 2017) was used for statistical analysis.

## Results

### Descriptive statistics

Demographic data in Table 1 shows a mean age (± standard deviation) of 38 (± 16) years of the sample. Among the interviewees, 51% were female, 49% were married, and 21% of the interviewees had at least one chronic disease. For educational attainment, one-third of the interviewees had a high school diploma, one-third of them had a bachelor’s degree, and about 28% of them received less than a high school education.

**Table 1:** Baseline characteristics of interviewees in NHIS-Taiwan (*n*=17,260)

	Mean	SD	Min	Max
Age (yrs)	38.12	15.94	12.01	65.00
Sex	Male: 48.92%, Female: 51.08%			
Marital status	Married: 48.91%, Separated: 2.25%, Divorced: 4.76%, Widowed: 2.72%, Never married: 41.15%			
Education	High school diploma: 32.81%, Bachelor's degree: 33.82%, Graduate school education: 5.21%, Less than a high school education: 28.11%			
Chronic Disease(s)	Yes: 21.32% (3,677), No: 78.68% (13,566)			
For those who had chronic disease(s)				
Have ever had the disease	Hypertension	Yes: 41.63% (1,530), No: 58.37% (2,145)		
	Diabetes	Yes: 19.94% (732), No: 80.06% (2939)		
	Dyslipidemia	Yes: 33.59% (1222), No: 66.41% (2416)		
	Stroke	Yes: 3.75% (138), No: 96.25% (3538)		
	Asthma	Yes: 6.90% (254), No: 93.06% (3422)		
	Chronic kidney disease	Yes: 9.60% (352), No: 90.40% (3316)		
Have had the disease during the past year^a^	Heart disease	Yes: 11.62% (427), No: 88.38% (3248)		
	Gout	Yes: 8.89% (327), No: 91.11% (3350)		
	Peptic ulcer	Yes: 9.68% (356), No: 90.29% (3320)		
	COPD	Yes: 2.31% (85), No: 97.69% (3591)		
	Liver/gall bladder disease	Yes: 13.63% (501), No: 86.37% (3174)		
	Osteoporosis	Yes: 12.33% (453), No: 87.67% (3221)		
	Cancer	Yes: 3.24% (119), No: 96.76% (3558)		
	Osteoarthritis	Yes: 9.28% (341), No: 90.72% (3332)		
	Psychiatric disorder	Yes: 8.82% (324), No: 91.18% (3350)		
	BPH (male)	Yes: 7.55% (153), No: 92.45% (1874)		
	Uterine/ovarian disease (female)	Yes: 7.55% (124), No: 92.45% (1519)		

^a^*COPD* Chronic obstructive pulmonary disease, *BPH* Benign prostatic hyperplasia

The first five items in EQ-5D all have a mean close to one (D1: 1.02±0.13; D2: 1.01±0.10; D3: 1.02±0.15; D4: 1.10±0.32; D5: 1.04±0.22) on a scale of 1 to 3 and the sixth item has a mean of 79.95 (SD: 13.58) on a scale of 0 to 100. These numbers show our interviewees generally had a good health state. The first three items in EQ-5D (D1. Mobility, D2. Self-care, and D3. Usual activities) have a moderate correlation (range, 0.52-0.69) with each other. However, the last three items (D4. Pain/Discomfort, D5. Anxiety/Depression, and D6. Overall health) have a weak correlation (range, 0.12-0.31) with all the other items. Scores across the first 5 items of EQ-5D show moderate internal consistency with a Cronbach’s alpha of 0.60. This alpha value supports moderate reliability for scores.

The distributions of the scores of the first five items in EQ-5D are highly concentrated at 1. To enable the IRT graded response model to converge upon estimates, we dichotomized the first five items with the first choice scored as 1 and the second and third choices scored as 0. Most of the scores of the sixth item are in multiples of ten. We thus divided these scores into eleven parts (0 stands for scores less than 5; 1 stands for 5-15; … ; 10 stands for scores more than 95) for analysis.

### Dimensionality of EQ-5D scale

A 1-dimensional factor analytic model was fit to the data to determine whether items measured a single underlying latent dimension. Standardized factor loadings ranged from 0.27 (D5, D6) to 0.84 (D3). Model fit indices were within acceptable ranges (χ^2^= 1392.41, *df*= 9, root mean square error of approximation = 0.14, comparative fit index = 0.88, standardized root mean square residual = 0.09), indicating that a single common factor can account for the relationships among item responses. A principal components analysis showed that an ideally weighted composite of item scores accounts for 32% of total variation. A scree plot from this analysis indicated that the first component accounted for substantially more variation than subsequent composites, suggesting that EQ-5D was measuring a unidimensional ability (HRQoL).

### Estimation of IRT graded response model

**Fig. 1:**
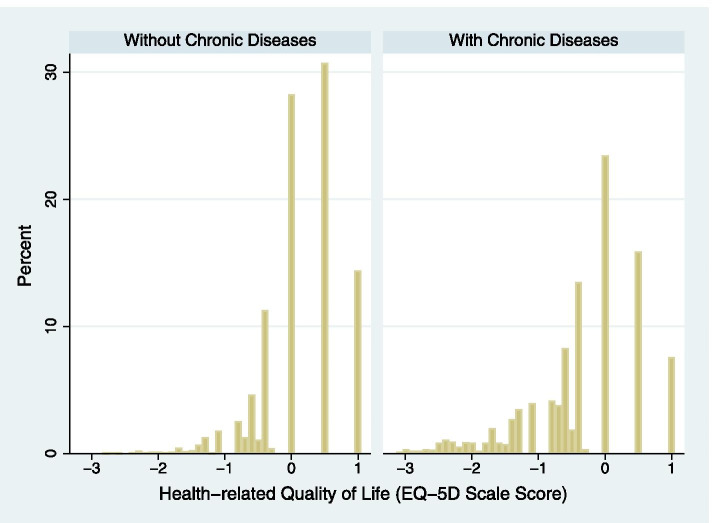
Histograms of the health-related quality of life (EQ-5D scale score) for groups with and without chronic diseases

An IRT graded response model was fit to the sample to estimate information and location parameters. In Table 2, the first five items all have a location parameter near -2 (range, -2.58 to -1.72). The first three items have high information parameters (range, 6.56-8.66), which indicate these three items could effectively distinguish people with very low HRQoL (e.g. below the 10th percentile). The EQ-5D scale score was estimated from the graded response model. We presented the EQ-5D scale score by dividing the sample into two groups: relatively healthy people and patients with chronic diseases (Fig. 1). We found there was about 10% of scale scores distributed around -2 in patients with chronic diseases while a scale score lower than -1.8 was rarely seen in relatively healthy people. To understand how each disease may impact on HRQoL, we presented EQ-5D scale scores for specific disease subgroups in Table 3. Stroke is the disease with the lowest scale score (-1.04), followed by psychiatric disorder (-0.91), COPD (-0.72), cancer (-0.70), and osteoarthritis (-0.69). Liver/gallbladder disease, gout, and uterine/ovarian disease (female) have the least impact on HRQoL, with scale scores ranged from -0.33 to -0.25.

**Table 2:** Parameters of the graded response model

Domain^a^	Information parameter	Location parameter
D1	6.56	-2.24
D2	7.44	-2.58
D3	8.66	-2.19
D4	1.94	-1.72
D5	1.98	-2.35
D6	1.49	-5.67, -4.70, -4.42, -4.05, -3.60, -2.61, -1.74, -0.91, 0.29, 1.70

^a^D1-D5 rescaled as 0 (for those who answered 2 or 3) and 1; D6 rescaled as 0-10 (0 stands for less than 5; 1 stands for 5-15; … ; 10 stands for more than 95)

**Table 3:** EQ-5D scale score for specific disease groups (from low to high)

Disease*	Mean (SD)		
Stroke	-1.04 (1.25)	**BPH (male)**	-0.45 (0.90)
Psychiatric disorder	-0.91 (0.93)	**Chronic kidney disease**	-0.41 (0.95)
COPD	-0.72 (0.94)	**Asthma**	-0.38 (0.82)
Cancer	-0.70 (0.98)	**Hypertension**	-0.37 (0.86)
Osteoarthritis	-0.69 (0.94)	**Peptic ulcer**	-0.37 (0.92)
Heart disease	-0.56 (0.92)	**Liver/gall bladder disease**	-0.33 (0.83)
Osteoporosis	-0.52 (0.95)	**Gout**	-0.32 (0.85)
Diabetes	-0.50 (0.87)	**Uterine/ovarian disease (female)**	-0.25 (0.82)
Hyperlipidemia	-0.49 (0.97)		

*COPD* Chronic obstructive pulmonary disease, *BPH* Benign prostatic hyperplasia

**Fig. 2:**
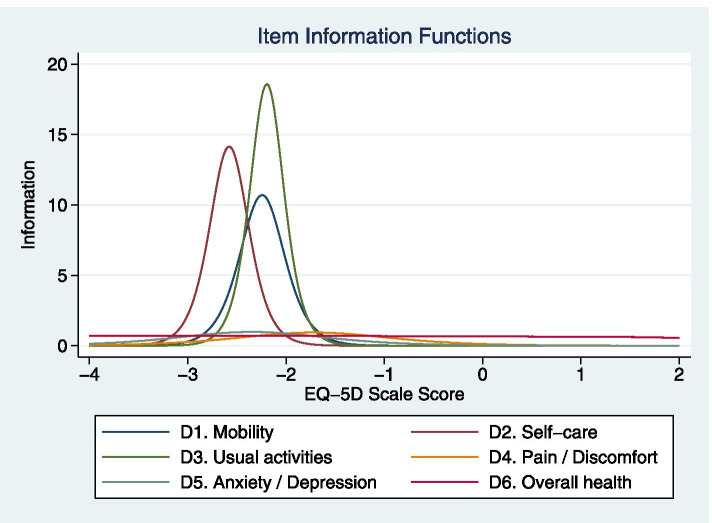
Item information function plots for 6 items in EQ-5D in the graded response model

The item information functions are presented (Fig. 2). The first three items provide much more information than the last three items do when the EQ-5D scale score is located around -2. The last three items provide equal information across all range of scale score. We then calculated the CSEMs for EQ-5D scale score in the graded response model. The CSEM is the lowest, around 0.2, when the scale score is located near -2, meaning that the precision is highest when we estimate EQ-5D scale score for people with very low HRQoL. The CSEM is high, around 1, when the scale score is located near 0, which means the precision in estimating EQ-5D scale score for people with the average HRQoL is low. Based on these results, we noted those patients with chronic diseases and HRQoL below the 10th percentile could be better differentiated by the first three items of EQ-5D than the other items. If differentiation is necessary for patients with average HRQoL, the other items may provide some information.

### Correlation analysis

To examine whether the EQ-5D scores can substitute for the SF-36 scores, we performed a correlation analysis for 8,272 interviewees who had both EQ-5D and SF-36 scores in our sample. In Table 4, the correlation is moderate between EQ-5D scores and SF-36 scores. Both the EQ-5D index (the average score of the first five items) and the EQ-5D scale score are moderately correlated with SF-36 PCS and SF-36 total score, with correlation coefficients of 0.61. However, the EQ-5D VAS (the sixth item) has a relatively weak correlation (r: 0.50) with SF-36 total score. The EQ-5D index, the EQ-5D VAS, and the EQ-5D scale score all have a lower correlation (r: 0.42-0.48) with SF-36 MCS.

**Table 4:** Correlation coefficients between EQ-5D and SF-36 scores (*n*=8,272)

	SF-36 Physical component score (PCS)	SF-36 Mental component score (MCS)	SF-36 Total score
**EQ-5D Index**	0.63	0.46	0.61
**95% CI**	0.62-0.65	0.44-0.48	0.60-0.62
**EQ-5D VAS**	0.47	0.42	0.50
**95% CI**	0.45-0.49	0.40-0.44	0.48-0.51
**EQ-5D Scale Score**	0.60	0.48	0.61
**95% CI**	0.59-0.62	0.47-0.50	0.59-0.62

## Discussion

According to our study, the correlation is moderate between EQ-5D scores and SF-36 scores. Using the IRT model, we found the EQ-5D scale score is moderately correlated with SF-36 PCS and SF-36 total score. Patients with stroke, psychiatric disorder, COPD, cancer, and osteoarthritis have a higher chance of impaired quality of life. The item information functions reveal that patients with chronic diseases and HRQoL below the 10th percentile could be better differentiated by the first three items of EQ-5D than the other items.

The EQ-5D scale has only 6 items, far fewer than 36 items of the SF-36 scale. For survey takers, it takes at least 10 minutes to complete the SF-36 scale, but it only takes less than 2 minutes to fill out the EQ-5D scale. The EQ-5D scale can bring potential benefits by saving time and money for the purpose of public health and clinical investigation. It is also of great importance that each country validates their own use of the EQ-5D scores, which will inform future practice in the local context. Using the IRT graded response model for quality of life research has been rarely seen in the previous literature in Taiwan, but it provides many insights into the analysis and interpretation of EQ-5D scores. Since our sample was representative of the national population in Taiwan, we can have an estimate of the average HRQoL using the EQ-5D scale score from the graded response model and establish norms for comparison in the future.

Our study shows that both the EQ-5D index (the average score of the first five items) and the EQ-5D scale score (the ability value estimated from the IRT model) have a moderate correlation with the SF-36 total scores. Although the EQ-5D index and the EQ-5D scale score share similar correlation coefficients with the external criterion, the EQ-5D scale score has more information, because the graded response model weights each item according to its information. The correlation coefficient between the EQ-5D index and the EQ-5D scale score is 0.70 (far from 0.99), supporting that the EQ-5D scale score is providing different information than the EQ-5D index does.

The information function from the IRT graded response model helps clarify which items are more informative at a specific range of the EQ-5D scale score. In our findings, three items, D1. Mobility, D2. Self-care, and D3. Usual activities, provide much more information for interviewees with an EQ-5D scale score near -2 than the other items do. According to the calculated CSEMs, the precision is highest when we estimate EQ-5D scale score for people with the scale score located near -2. The scale score of -2 is the 10th percentile of EQ-5D scale score in people with chronic diseases, while a scale score lower than -1.8 is rarely seen in relatively healthy people. Thus, for patients who have chronic diseases and an EQ-5D scale score below the 10th percentile, the first three items of the EQ-5D scale are useful to tell whether their quality of life is impaired (very low or low). Clinicians can have these three items as a set of screening questions if they encounter a patient with chronic diseases and suspected impaired quality of life. If the patient reports any decreased function in these three items, the clinician should arrange the corresponding management plan to improve (or at least maintain) the patient’s health state and quality of life.

One thing worth mentioning is that we have dichotomized the first five items of the EQ-5D scale and divided the scores of sixth item into eleven parts (0, 1, 2, … , 10) for the IRT graded response model. This version of scale showed concentrated information around a scale score of -2. Keeping the first three items of the EQ-5D scale with only 2 score points can be an even more efficient way and provide us adequate information to differentiate patients who have very low and low levels of quality of life. We suggest to use the 2-point scale in the clinic setting for its relative convenience.

The items of mobility (D1), self-care (D2), and usual activities (D3) had higher correlations between EQ-5D and SF-36. One possible explanation is that people in Taiwan less frequently reported problems in the first three items. Once reported, the problems were often severe and impaired their quality of life. A previous EQ-5D validation study in Taiwan showed similar results [17]. We further performed the same IRT technique for different age groups of our interviewees (groups aged 12-24, 25-44, and 45-64). We found the three items are the most informative items in all these groups, but with some differences in the pattern of item information functions. One significant difference in the item information functions is that mobility (D1) provides much more information than the rest two items do in the group aged 25-44. It reveals the fact that any impact on mobility can endanger the work and life of people in this age group and severely impaired their HRQoL.

In this NHIS dataset, we registered a variety of chronic diseases that were diagnosed by the physicians rather than simply reported by the interviewees. When examining the EQ-5D scale scores by disease subgroups, we found patients with different types of chronic diseases had different levels of HRQoL to various degrees. Patients with stroke, psychiatric disorder, COPD, cancer, and osteoarthritis have a higher chance of impaired quality of life. Clinicians need to be attentive to these subgroups of patients with chronic diseases. The EQ-5D scale can be a useful tool to assess whether the quality of life is impaired among the high-risk patient population.

Although IRT shows great benefits by revealing the item characteristics, there are some considerations when using the IRT graded response model. First, for polytomous items like EQ-5D VAS, a large sample size (above 3,500) and coverage across polytomous item scales are needed [19]. The case number in our sampling is large enough for us to fit the IRT model. Second, we can only gather information from the given items of our scale. If the goal is to find more details in each dimension of EQ-5D, an in-depth survey with more items is needed. According to our correlation analysis, the EQ-5D scale score itself is a moderate predictor for the SF-36 score. The finding supports that the EQ-5D scale could be a useful and efficient alternative of SF-36 to quickly screen patients’ HRQoL under time constraints. However, if we want to understand how patients with different diseases have different quality of life, it is vital to examine the EQ-5D scores for each type of chronic disease and link them to scores of other HRQoL measures with more items in the following studies.

The EQ-5D scores in our study demonstrate a higher correlation with the SF-36 physical component score than with the mental component score. Some diseases are known to be highly associated with mental health problems and may show a different pattern of information function of EQ-5D items in the IRT graded response model if the sample targets the population of people with these diseases. Similar issues have been raised in a previous review of psychometrics and qualitative assessment of EQ-5D [15]. Therefore, future research using IRT is needed to understand how to interpret the scores of EQ-5D items for patients with specific diseases and across different clinical settings.

## Conclusions

Use of the EQ-5D scale scores is appropriate in the general population, particularly for distinguishing between patients who have very low and low HRQoL. The EQ-5D scores have moderate internal reliability and moderate correlation with SF-36 scores. The IRT graded response model strengthens our interpretation of the EQ-5D scores. The information function analysis demonstrates that Domain 1 (Mobility), Domain 2 (Self-care) and Domain 3 (Usual activities) are the three most informative items of the EQ-5D scale for patients who have chronic diseases and HRQoL below the 10th percentile. Subgroup analysis shows that patients with stroke, psychiatric disorder, COPD, cancer, and osteoarthritis have a higher chance of impaired quality of life. If the time constraints in clinical settings are severe and efficient distinction between very low and low HRQoL patients is desired, we suggest using EQ-5D instead of SF-36 to measure the HRQoL for patients with chronic diseases.

## Supplementary Information

**Additional file 1.**

## Data Availability

The data that support the findings of this study are available from National Health Research Institute, Taiwan. Restrictions apply to the availability of these data, which were used under license for this study. Data are available from the authors with the permission of National Health Research Institute, Taiwan.

## Abbreviations

HRQoL: Health-related quality of life
EQ-5D: EuroQoL 5-dimension
SF-36: 36-Item Short Form Survey
IRT: Item response theory
NHIS: National Health Interview Survey
PPS: Probability proportional to size
COPD: Chronic obstructive pulmonary disease
BPH: Benign prostatic hyperplasia
VAS: Visual analogue scale
PCS: Physical component score
MCS: Mental component score
CSEM: Conditional standard error of measurement
