# Supplementary material for: Validation of the EQ-5D in Taiwan using item response theory
Source: BMC Public Health. 2021 Dec 19;21:2305. doi: 10.1186/s12889-021-12334-y (PMC8684691; doi:10.1186/s12889-021-12334-y)
Supplement: Supplementary file 1 — Additional file 1. [file 12889_2021_12334_MOESM1_ESM.docx]

**Supplementary material A. EQ-5D in the NHIS-Taiwan (2013)**

Under each heading, please tick the ONE box that best describes your health TODAY.

1. **MOBILITY**

1 - I have no problems in walking about

2 - I have some problems in walking about

3 - I am confined to bed

1. **SELF-CARE**

1 - I have no problems with self-care

2 - I have some problems washing or dressing myself

3 - I am unable to wash or dress myself

1. **USUAL ACTIVITIES** (e.g. work, study, housework, family or leisure activities)

1 - I have no problems with performing my usual activities

2 - I have some problems with performing my usual activities

3 - I am unable to perform my usual activities

1. **PAIN / DISCOMFORT**

1 - I have no pain or discomfort

2 - I have moderate pain or discomfort

3 - I have extreme pain or discomfort

1. **ANXIETY / DEPRESSION**

1 - I am not anxious or depressed

2 - I am moderately anxious or depressed

3 - I am extremely anxious or depressed

1. **OVERALL HEALTH**

The best health you can imagine

**
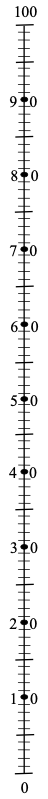
**We draw a scale (pretty much like a thermometer) to help
people express whether their health is good or bad.
On this scale, 100 means the best health you can imagine,
and 0 means the worst health you can imagine.

We hope that you mark an X on the scale to indicate how
your health is TODAY based on your thoughts. You can
draw a line from the box below and link it to a point on the
scale representing how good or bad your health is TODAY.

SCORE:

YOUR HEALTH TODAY

The worst health you can imagine

**Supplementary material B. SF-36 in the NHIS-Taiwan (2013)**

**1. In general, would you say your health is:**

1 - Excellent 2 - Very good 3 - Good 4 - Fair 5 - Poor

**2. Compared to one year ago, how would you rate your health in general now?**

1 - Much better now than one year ago 2 - Somewhat better now than one year ago 3 - About the same 4 - Somewhat worse now than one year ago 5 - Much worse now than one year ago

The following items are about activities you might do during a typical day. Does your health now limit you in these activities? If so, how much?

**3. Vigorous activities, such as running, lifting heavy objects, participating in strenuous sports**

1- Yes, limited a lot 2- Yes, limited a little 3- No, not limited at all

**4. Moderate activities, such as moving a table, mopping the floor, bowling, or playing taichi**

1- Yes, limited a lot 2- Yes, limited a little 3- No, not limited at all

**5. Lifting or carrying groceries**

1- Yes, limited a lot 2- Yes, limited a little 3- No, not limited at all

**6. Climbing several flights of stairs**

1- Yes, limited a lot 2- Yes, limited a little 3- No, not limited at all

**7. Climbing one flight of stairs**

1- Yes, limited a lot 2- Yes, limited a little 3- No, not limited at all

**8. Bending, kneeling, or stooping**

1- Yes, limited a lot 2- Yes, limited a little 3- No, not limited at all

**9. Walking more than a kilometer**

1- Yes, limited a lot 2- Yes, limited a little 3- No, not limited at all

**10. Walking several blocks**

1- Yes, limited a lot 2- Yes, limited a little 3- No, not limited at all

**11. Walking one block**

1- Yes, limited a lot 2- Yes, limited a little 3- No, not limited at all

**12. Bathing or dressing yourself**

1- Yes, limited a lot 2- Yes, limited a little 3- No, not limited at all

During the past one month, have you had any of the following problems with your work or other regular daily activities as a result of your physical health?

**13. Cut down the amount of time you spent on work or other activities**

1- Yes 2- No

**14. Accomplished less than you would like**

1- Yes 2- No

**15. Were limited in the kind of work or other activities**

1- Yes 2- No

**16. Had difficulty performing the work or other activities (for example, it took extra effort)**

1- Yes 2- No

During the past one month, have you had any of the following problems with your work or other regular daily activities as a result of any emotional problems (such as feeling depressed or anxious)?

**17. Cut down the amount of time you spent on work or other activities**

1- Yes 2- No

**18. Accomplished less than you would like**

1- Yes 2- No

**19. Didn't do work or other activities as carefully as usual**

1- Yes 2- No

**20. During the past one month, to what extent has your physical health or emotional problems interfered with your normal social activities with family, friends, neighbors, or groups?**

1 - Not at all 2 - Slightly 3 - Moderately 4 - Quite a bit 5 - Extremely

**21. How much bodily pain have you had during the past one month?**

1 - None 2 - Very mild 3 - Mild 4 - Moderate 5 - Severe 6 - Very severe

**22. During the past one month, how much did pain interfere with your normal work (including both work outside the home and housework)?**

1 - Not at all 2 - Slightly 3 - Moderately 4 - Quite a bit 5 - Extremely

These questions are about how you feel and how things have been with you during the past one month. For each question, please give the one answer that comes closest to the way you have been feeling.

How much of the time during the past one month...

**23. Did you feel full of pep?**

1 - All of the time 2 - Most of the time 3 - A good bit of the time 4 - Some of the time 5 -A little of the time 6 - None of the time

**24. Have you been a very nervous person?**

1 - All of the time 2 - Most of the time 3 - A good bit of the time 4 - Some of the time 5 -A little of the time 6 - None of the time

**25. Have you felt so down in the dumps that nothing could cheer you up?**

1 - All of the time 2 - Most of the time 3 - A good bit of the time 4 - Some of the time 5 -A little of the time 6 - None of the time

**26. Have you felt calm and peaceful?**

1 - All of the time 2 - Most of the time 3 - A good bit of the time 4 - Some of the time 5 -A little of the time 6 - None of the time

**27. Did you have a lot of energy?**

1 - All of the time 2 - Most of the time 3 - A good bit of the time 4 - Some of the time 5 -A little of the time 6 - None of the time

**28. Have you felt downhearted and blue?**

1 - All of the time 2 - Most of the time 3 - A good bit of the time 4 - Some of the time 5 -A little of the time 6 - None of the time

**29. Did you feel worn out?**

1 - All of the time 2 - Most of the time 3 - A good bit of the time 4 - Some of the time 5 -A little of the time 6 - None of the time

**30. Have you been a happy person?**

1 - All of the time 2 - Most of the time 3 - A good bit of the time 4 - Some of the time 5 -A little of the time 6 - None of the time

**31. Did you feel tired?**

1 - All of the time 2 - Most of the time 3 - A good bit of the time 4 - Some of the time 5 -A little of the time 6 - None of the time

**32. During the past one month, how much of the time has your physical health or emotional problems interfered with your social activities (like visiting with friends, relatives, etc.)?**

1 - All of the time 2 - Most of the time 3 - Some of the time 4 - A little of the time 5 - None of the time

How TRUE or FALSE is each of the following statements for you.

**33. I seem to get sick a little easier than other people**

1 - Definitely true 2 - Mostly true 3 - Don't know 4 - Mostly false 5 - Definitely false

**34. I am as healthy as anybody I know**

1 - Definitely true 2 - Mostly true 3 - Don't know 4 - Mostly false 5 - Definitely false

**35. I expect my health to get worse**

1 - Definitely true 2 - Mostly true 3 - Don't know 4 - Mostly false 5 - Definitely false

**36. My health is excellent**

1 - Definitely true 2 - Mostly true 3 - Don't know 4 - Mostly false 5 - Definitely false
